# Empirical Evidence That Complexity Limits Horizontal Gene Transfer

**DOI:** 10.1093/gbe/evad089

**Published:** 2023-05-26

**Authors:** Christina L Burch, Artur Romanchuk, Michael Kelly, Yingfang Wu, Corbin D Jones

**Affiliations:** Department of Biology, University of North Carolina, Chapel Hill, North Carolina, USA; Department of Biology, University of North Carolina, Chapel Hill, North Carolina, USA

**Keywords:** evolution, experiment, bacteria, genetic exchange, recombination

## Abstract

Horizontal gene transfer (HGT) is a major contributor to bacterial genome evolution, generating phenotypic diversity, driving the expansion of protein families, and facilitating the evolution of new phenotypes, new metabolic pathways, and new species. Comparative studies of gene gain in bacteria suggest that the frequency with which individual genes successfully undergo HGT varies considerably and may be associated with the number of protein–protein interactions in which the gene participates, that is, its connectivity. Two nonexclusive hypotheses have emerged to explain why transferability should decrease with connectivity: the complexity hypothesis (Jain R, Rivera MC, Lake JA. 1999. Horizontal gene transfer among genomes: the complexity hypothesis. Proc Natl Acad Sci U S A. 96:3801–3806.) and the balance hypothesis (Papp B, Pál C, Hurst LD. 2003. Dosage sensitivity and the evolution of gene families in yeast. Nature 424:194–197.). These hypotheses predict that the functional costs of HGT arise from a failure of divergent homologs to make normal protein–protein interactions or from gene misexpression, respectively. Here we describe genome-wide assessments of these hypotheses in which we used 74 existing prokaryotic whole genome shotgun libraries to estimate rates of horizontal transfer of genes from taxonomically diverse prokaryotic donors into *Escherichia coli*. We show that 1) transferability declines as connectivity increases, 2) transferability declines as the divergence between donor and recipient orthologs increases, and that 3) the magnitude of this negative effect of divergence on transferability increases with connectivity. These effects are particularly robust among the translational proteins, which span the widest range of connectivities. Whereas the complexity hypothesis explains all three of these observations, the balance hypothesis explains only the first one.

**Significance**

Comparisons between prokaryotic genomes consistently show that genes with informational functions, for example, in genome replication, transcription, and translation, have been subject to horizontal gene transfer between species more often than genes with operational functions, for example, in metabolism and environmental sensing. In this study, we perform a genome-wide analysis of transferability, using data obtained from 74 genomes, to show that this pattern results from differences between informational and operational genes in the number of other proteins with which they interact, that is, their connectivity, rather than from their functional differences. Our analysis underscores the need for an exceptionally large data set to detect connectivity effects on transferability, explaining why past experimental studies failed to replicate the consistent finding from comparative genomic studies.

## Introduction

Horizontal gene transfer (HGT) is a major contributor to bacterial genome evolution (Skippington and Ragan 2011; Brito 2021; Arnold et al. 2022), contributing 10–20% of the protein-coding genes to most bacterial genomes (Lawrence and Ochman 1998; Nakamura et al. 2004; Soucy et al. 2015). HGT promotes diversity (Guttman 1997; Baltrus et al. 2011; Polz et al. 2013), facilitating the evolution of novel phenotypes (Moran and Jarvik 2010), metabolic pathways (Soyer and Creevey 2010), and species (Schaack et al. 2010). Numerous instances of rapid adaptation have been attributed to HGT (e.g., Lozupone et al. 2008; Dhillon et al. 2015; Frazão et al. 2019; Arnold et al. 2020; Woods et al. 2020; reviewed in Arnold et al. 2022).

Comparative studies reveal that the frequency of HGT varies among genes and among pairs of donor and recipient species in predictable ways (Nakamura et al. 2004; Pál et al. 2005; Soucy et al. 2015). For instance, transferability is observed to depend on gene function (Rivera et al. 1998; Nakamura et al. 2004), connectivity (i.e., the number of protein–protein interactions [PPIs]) (Lercher and Pál 2008; Cohen et al. 2011), and the divergence between donor and recipient genomes (Tuller et al. 2011; Baltrus 2013; Soucy et al. 2015).

**Fig. 1.**
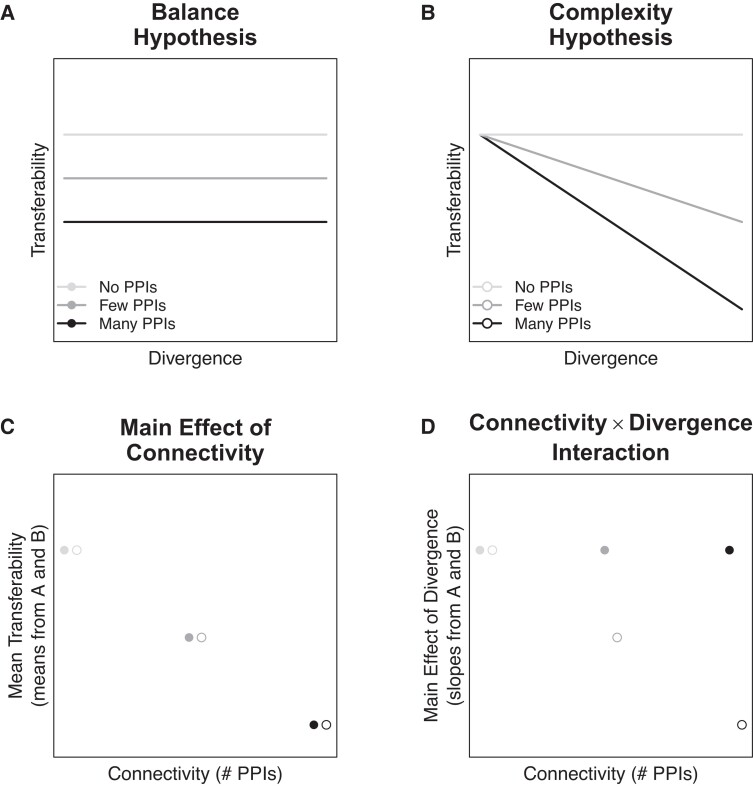
Central predictions of the complexity and balance hypotheses. The balance hypothesis (*A*) posits that transferred genes with resident orthologs may cause an imbalance in the expression of genes that participate in multiprotein complexes and must be produced in stoichiometric amounts. The cost of expression imbalance is predicted to increase with the number of PPIs, but not with divergence. As a result, genes that engage in PPIs (dark gray and black lines) are less transferable than genes that lack PPIs (light gray line). The complexity hypothesis (*B*) posits that transferred genes may interfere in the normal PPIs of the resident ortholog, and that the probability of interference increases with the divergence between the resident and transferred genes. As a result, transferability decreases with divergence, but only for genes that engage in PPIs (dark gray and black lines). Two testable predictions result from these hypotheses. The central prediction of the balance hypothesis (*C*) is that the transferability of a particular gene, averaged over all donor orthologs, should decrease as the connectivity of the gene increases. In statistical lingo, the balance hypothesis predicts a *main effect of connectivity*. Points in (*C*) are mean values of lines of the same shade in (*A*) and (*B*), illustrating that this prediction does not distinguish between the balance (filled circles) and complexity (open circles) hypotheses. The central prediction of the complexity hypothesis (*D*) is that the effect of divergence on transferability (i.e., the *main effect of divergence*) should become more negative as connectivity increases. In statistical lingo, the complexity hypothesis predicts a *connectivity* × *divergence interaction*. Points in (*D*) are slopes of lines of the same shade in (*A*) and (*B*), illustrating that only the complexity hypothesis predicts a connectivity × divergence interaction, shown here as a decreasing main effect of divergence from light gray to dark gray to black, among the open cirles. The balance hypothesis makes no such prediction: the main effect of divergence does not differ between the filled circles.

Two nonexclusive hypotheses have been proposed to explain why the fitness cost of gene transfer increases with connectivity: the balance hypothesis (Papp et al. 2003) and the complexity hypothesis (Jain et al. 1999). These hypotheses predict that the fitness costs of HGT arise from gene misregulation (balance hypothesis) or from the failure of transferred orthologs to engage in normal PPIs (complexity hypothesis). The central predictions of these hypotheses are illustrated in figure 1. Whereas costs associated with gene misregulation are expected regardless of the divergence between the resident and transferred orthologs (fig. 1*A* and *C*), costs associated with PPI failure are expected to increase in frequency with divergence (fig. 1*B* and *D*). In both hypotheses, the average magnitude of the fitness cost is expected to increase with connectivity.

Because comparative genomic data often rely on the existence of sequence divergence to detect HGT, comparative data can document only cases where gene transfer had the potential to affect both gene regulation and PPIs. HGT of similar or identical alleles, expected to result in gene misregulation but not PPI failure, would not be detectable by comparative methods. Thus, experimental data are needed to distinguish between these two effects. To date, experimental data have confirmed that the fitness costs of HGT vary among genes (Sorek et al. 2007; Knöppel et al. 2014; Acar Kirit et al. 2020) and depend on the divergence between donor and recipient genomes (Sorek et al. 2007). However, previous experimental tests have failed to detect an effect of connectivity on transferability (Acar Kirit et al. 2020).

Here we use a genome-wide quantitative approach to provide a direct test of the complexity and balance hypotheses. Following Sorek et al. (2007), we analyze the data generated during whole genome shotgun sequencing of 70 bacterial and 4 archaeal genomes. In the shotgun approach, random genome fragments were sequenced only after they were successfully cloned into a plasmid and transformed (i.e., transferred) into *Escherichia coli*. Thus, genes that imposed fitness costs as a result of their incidental expression from the plasmid may have been underrepresented in the shotgun libraries. Sorek et al. (2007) investigated *qualitative* differences in gene representation (presence or absence) in the shotguns to explain the inability of some specific genes from some specific species to transfer into *E. coli*. They confirmed experimentally 1) that most genes in the libraries were expressed after transfer to *E. coli* and 2) that their expression was a critical factor in the absence of certain genes from the libraries. They also confirmed that 3) the genes’ high copy number on multicopy cloning plasmids (Chang and Cohen 1978; Summers 1998) was not the sole determinant of their transferability, as evidenced by the finding that genes that were absent from the shotgun libraries tended to be untransferable on single-copy fosmids, as well (Sorek et al. 2007). We build on their analysis by investigating *quantitative* differences in gene representation in the shotgun sequencing data, to test general hypotheses for differences in transferability among genes and genomes (as in Knöppel et al. 2014; Acar Kirit et al. 2020). We test the complexity and balance hypotheses by investigating the effects of connectivity and divergence on the number of times individual genes, in their entirety, were successfully cloned, transformed, and sequenced in the whole genome shotguns. The strong statistical power of our genome-wide quantitative approach enabled detection of both a main effect of connectivity and of an interaction effect between connectivity and divergence on the transferability (representation in the shotgun data) of individual genes, confirming the central predictions of the balance and complexity hypotheses, respectively (fig. 1).

## Results

**Fig. 2.**
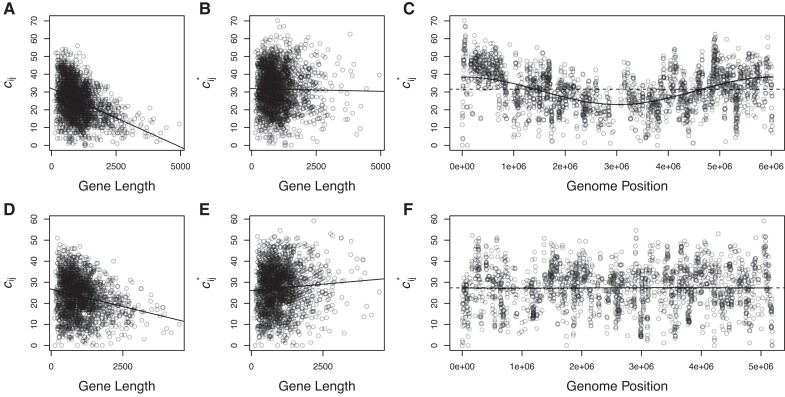
Methodological effects on coverage (*c_ij_*) in whole genome shotguns. As shown here for *P. syringae* (*A*) and *Pseudoalteromonas atlantica* (*D*), long genes are substantially less well covered than short genes in all shotguns. By adjusting the raw coverage values of each gene solely by the likelihood of complete coverage given the gene's length (to obtain c_ij_*; see Materials and Methods), the bias against long genes is dramatically reduced, as shown here for *P. syringae* (*B*) and *P. atlantica* (*E*). These adjusted coverage values, c_ij_*, show positional biases in some genomes, for example, *P. syringae* (*C*), but not others, for example, *P. atlantica* (*F*). In all panels, points represent the coverage or adjusted coverage of individual genes (i.e., coding sequences) in the *P. syringae* (*A*–*C*) or *P. atlantica* (*D*–*F*) shotgun. Solid black lines (*A*, *B*, *D*, *E*) show the best fit linear relationship between coverage or adjusted coverage and gene length. Dashed lines (*C*, *F*) are the mean adjusted coverage across all genes in the individual shotguns. Solid lines (*C*, *F*) are the sine curves that yield the best fit to the adjusted coverage data. The solid and dashed lines in *F* are indistinguishable. Supplementary fig. S1, Supplementary Material online, shows analogous data and model fits for the complete set of genome shotguns.

We acquired the shotgun library sequences of 74 prokaryotic species (70 bacteria and 4 archaea, described in supplementary table S1, Supplementary Material online) from the NCBI Trace Archive, following Sorek et al. (2007). We then calculated the coverage of each coding sequence in each shotgun library as the number of plasmid inserts in the library that contained the gene in its entirety (as described in Materials and Methods: Gene Coverage in the Shotguns). A visual examination of the coverage variation among genes within individual shotgun libraries (figs. 2 and S1, Supplementary Material online) revealed two general patterns: 1) long genes are less well covered than short genes in all shotguns and 2) genes near the origin of replication are better covered than genes near the terminus of replication, but to different degrees in different shotguns. These patterns suggested strong effects of the particular pretransformation methods used to generate the shotgun libraries; thus, we first determined the extent to which variance in the gene coverage within libraries could be explained by the particular methods used to generate the libraries. Below, we first describe how the shotgun library methods were likely to bias gene coverage and how we controlled for these pretransformation methodological effects on variance in coverage. We then describe our investigation of how biological features—such as connectivity, divergence from the *E. coli* ortholog, and their statistical interaction—explain the remaining variance in coverage; variance that we infer to have resulted from differences in transformation efficiency, that is, transferability.

### Effect of Gene Length on Coverage

In figures 2*A*, 2*D*, and S1, Supplementary Material online, we show the relationship between coverage and gene length in each shotgun library (note that two species possess two chromosomes and we analyzed data for each chromosome separately). It is apparent from these figures that long genes are less well covered than short genes in all shotgun libraries. The likely reason is straightforward. Long genes are less likely to be entirely contained within short cloned fragments.

To correct for this bias against long genes, we calculated the expected bias, *b_ij_*, against each gene *i* from genome *j*, given the length of the gene, *L_ij_*, and the distribution of cloned fragment lengths from genome *j* (supplementary fig. S1, Supplementary Material online; details in Materials and Methods: Effect of Gene Length on Coverage). We then divided the observed raw coverage value, *c_ij_*, by the expected bias, *b_ij_*, to obtain an unbiased, length-corrected estimate c_ij_* for that gene (figs. 2*B*, 2*E*, and S1, Supplementary Material online). Before correcting for length bias, the effect of gene length on coverage was negative for 74 out of 74 bacterial chromosomes (supplementary fig. S1, Supplementary Material online). After correcting for length bias, the effect of gene length on coverage was negative in 21 and positive in 53 shotguns (the unbiased expectation is 37 negative and 37 positive) and has a much smaller statistical effect on coverage. Whereas gene length explains up to 43% of variance in raw coverage within individual shotgun libraries (median *R*^2^ = 0.092; range = 0.0023–0.43), gene length explains only up to 1.8% of variance in length-adjusted coverage (median *R*^2^ = 0.0020; range = 1.0 × 10^−5^–0.036).

### Effect of Gene Position on Coverage

In figures 2*C*, 2*F*, and S1, Supplementary Material online, we show the adjusted coverage values, c_ij_*, plotted against position in the genome for individual shotguns. The most obvious pattern is that genes near the origin of replication had higher coverage residuals than genes near the terminus of replication in *some* shotguns. Again, the likely reason for this pattern is straightforward. Because bacterial genome replication begins at the origin, genes near the origin are replicated before genes near the terminus. Thus, in actively dividing cells, genes near the origin are present in more copies within the cell than genes near the terminus. We infer that the difference in this pattern between shotguns resulted from a methodological difference in the bacterial growth phase, exponential or stationary, at the time genomes were harvested for use in the shotgun.

We estimated the positional bias in each genome *j* by fitting a sine curve to the c_ij_* values as a function of the start position of each gene *i* (solid red lines in figs. 2*C*, 2*F*, and S1, Supplementary Material online). The sine curves explained between 0.007% (fig. 2*F*; *Pseudomonas syringae* pv. *syringae*) and 45% (supplementary fig. S1, Supplementary Material online; *Arthrobacter* sp. fb24) of the variance in c_ij_* (interquartile range = 1.32–7.57% variance explained). To correct for this positional bias, we divided the c_ij_* values by the coverage expectation given the position of gene *i* in genome *j*, *E*(c_ij_* | position*_ij_*), obtained from the best fit sine curve. For all downstream analyses, we consider the effects of various independent variables on the resulting dependent variable ${\hat{c}}_{ij}$ = c_ij_*/*E*(c_ij_* | position*_ij_*), which we refer to as relative coverage.

### Biological Effects on Coverage

**Fig. 3.**
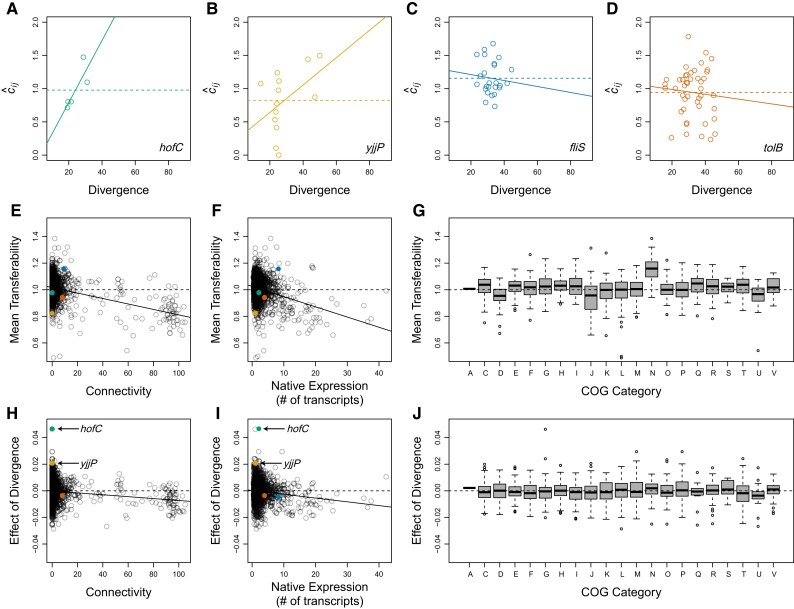
Biological effects on transferability in whole genome shotguns. We first estimated the mean value and the effect of divergence (% amino acid difference) on transferability among the set of orthologs of each protein-coding gene in the *E. coli* K12 genome. Estimates for individual genes are illustrated here for the *E. coli* genes *hofC* (*A*, green), *yjjP* (*B*, gold), *fliS* (*C*, blue), and *tolB* (*D*, orange) that are representative, respectively, of genes in the 1st, 25th, 50th, and 75th percentiles, in terms of their number of likely orthologs. For each gene, we estimated mean transferability as mean relative coverage (mean ${\hat{c}}_{ij}$; dashed colored lines in *A–D*), and we estimated the effect of divergence on transferability as the slope of the best fit linear relationship between relative coverage (${\hat{c}}_{ij}$) and divergence from the *E. coli* gene (solid colored lines in *A–D*). We then examined the effects of connectivity, native expression level, and COG functional category on the resulting estimates of mean transferability (*E*–*G*) and on the effect of divergence on transferability (*H*–*J*). Open colored circles in (*A*)–(*D*) show the relative coverage and divergence from the *E. coli* gene of likely orthologs of the *E. coli* gene shown on the corresponding plot. Each open colored circle in (*A*)–(*D*) was estimated from a different genome shotgun library. Filled colored points in (*E*), (*F*), (*H*), and (*I*) show the mean transferability and effect of divergence estimated from the gene in (*A*)–(*D*) with the corresponding color. Each open black circle in panels (*E*), (*F*), (*H*), and (*I*) shows the mean transferability and effect of divergence estimated from an individual gene with at least 16 likely orthologs and a standard deviation, among the orthologs, of greater than 10% amino acid difference. The genes *hofC* and *yjjP* are labelled in *H* and *I* to highlight that genes with few identified orthologs tended to yeild extreme estimates for the effect of divergence on transferability.

We next examined the effect of amino acid divergence between donor and recipient copies of orthologous transferred genes on relative coverage in the shotgun libraries. For each protein-coding gene in the *E. coli* K12 genome, we identified likely orthologs within the 74 shotgun libraries (see Effect of Divergence on Coverage). There was wide variation among protein-coding genes, in both the number of shotgun libraries that contained a likely ortholog and the divergence (% amino acid difference) of those orthologs from the *E. coli* copy of the gene (supplementary fig. S2, Supplementary Material online). For each gene, we then calculated mean relative coverage among its likely orthologs and the slope of the relationship between relative coverage and divergence. A visual examination of these data for different genes revealed dramatically higher variance in the estimated relationship (i.e., slope) between divergence and relative coverage among genes with fewer orthologs than among genes with more orthologs (figs. 3*A*–3*D* and S2, Supplementary Material online). For example, compare the genes shown in figure 3*A*–*D*, which are representative of genes from the 1st (*hofC*, fig. 2*A*, green), 25th (*yjjP*, fig. 2*B*, yellow), 50th (*fliS*, fig. 2*C*, blue), and 75th (*tolB*, fig. 2*D*, orange) percentiles, in terms of their number of likely orthologs. *hofC* and *yjjP* were randomly chosen from among genes with 5 and 13 likely orthologs, respectively, but, like other genes with few likely orthologs, the estimated effect of divergence on transferability for these genes (solid green and yellow circles in fig. 3*H* and *I*) is extreme compared with genes with many likely orthologs (open black circles in fig. 3*H* and *I*).

To reduce this source of noise in the data, we limited the analyses below to protein-coding genes in the *E. coli* K12 genome for which we identified likely orthologs in a reasonably large number of bacterial genomes and for which the likely orthologs spanned a reasonably wide range of divergence values from the *E. coli* gene. Specifically, we required that each set of orthologous genes include at least 16 orthologs and exhibit a standard deviation, among the orthologs, of greater than 10% amino acid difference. These criteria were chosen based on a power analysis of the entire data set (supplementary figs. S3 and S4, Supplementary Material online; described in Materials and Methods: Power Analysis) and reduced the data set to 1,295 sets of orthologous genes. However, the results we present below were not quantitatively sensitive to the choice of criteria (described in detail below). Thus, we proceeded with the set of 1,295 genes for which we have relatively high confidence in our estimates of both mean relative coverage and the effect of divergence on relative coverage. We investigated the effects of three biological characteristics of genes—connectivity, the expression level of the native gene in *E. coli*, and gene function as identified in the Clusters of Orthologous Groups of proteins (COG) database (Tatusov et al. 1997; Galperin et al. 2015)—on both of these estimates.

**Fig. 4.**
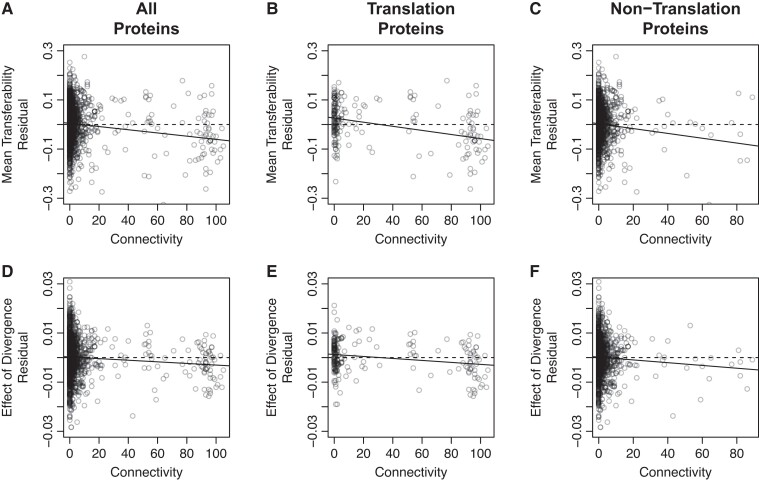
Tests of the balance and connectivity hypotheses. Plots show the mean transferability (*A*–*C*) and effect of divergence on transferability (*D*–*F*), after first controlling for the effects of native expression level and COG functional category. Each point shows the deviation (i.e., residual) of each gene’s value from the expected value given the native expression level and COG category of that gene. The different plots show all the genes in our collection (*A*, *D*), the translation proteins only (*B*, *E*), or all the genes except the translation proteins (*C*, *F*). Solid lines are best fit linear regression models. Dashed horizontal lines at zero are shown only for reference.

For a particular set of orthologous genes, mean relative coverage (horizontal dashed lines in figs. 3*A*–3*D* and S2, Supplementary Material online) provides a measure of the average transferability of divergent copies of that ortholog. Among the most stringent set of 1,295 protein-coding genes in our data set, all three biological characteristics had significant effects on this metric of transferability (table 1). Increasing connectivity and increasing expression level both had significant negative effects on mean transferability (fig. 3*E* and *F*), and there was significant variation among functional categories in their mean transferability (fig. 3*G*). Because these three biological characteristics are correlated with each other (supplementary fig. S5, Supplementary Material online), we determined the effects of connectivity, in isolation from the other two characteristics, by examining the deviations (i.e., residuals) of each gene's mean transferability from the value that would be expected given the gene's native expression level and COG functional category. We found a significant negative relationship between connectivity and the resulting mean transferability residuals (fig. 4*A*; effect estimate = −6.5 × 10^−4^, *F*_1,1294_ = 27.5, *P* = 2 × 10^−7^). Repeating the analysis using only the 192 genes involved in translation (COG category J; the subset of informational genes with the largest number of highly connected genes) reveals a strong contribution of these genes to the statistical power of the analysis. Nonetheless, the effect of connectivity on mean transferability appears to be a general characteristic of all protein-coding genes, not just informational genes. The effect is negative and of similar magnitude among the 192 translation proteins (fig. 4*B*; connectivity effect estimate = −8.3 × 10^−4^, *F*_1,191_ = 19.88, *P* = 1.4 × 10^−5^) and the 1,102 nontranslation proteins (fig. 4*C*; connectivity effect estimate = −9.8 × 10^−4^, *F*_1,1102_ = 10.54, *P* = 0.0012). These effect estimates and *P* values were quantitatively similar and qualitatively unchanged in analyses of the 2,653 (or 1,883) orthologous gene sets that met the less stringent criteria of including a minimum of 4 (or 10) orthologs and a standard deviation greater than 4% (or 7%) amino acid difference (supplementary table S2, Supplementary Material online).

**Table 1:** Analysis of Variance Table: Biological Effects on Mean Transferability

Model Parameter	df	SSE	MSE	*F* Value	*P* Value
Connectivity	1	2.2312	2.23123	274.1	2.2 × 10^−16^
COG functional category	20	1.0647	0.05324	6.542	2.2 × 10^−16^
Native expression level	1	0.1656	0.16555	20.34	7.1 × 10^−6^
Residuals	1,273	10.359	0.00814	—	—

Note.—Statistical model: mean transferability ∼ connectivity + COG category + native expression level.

For a particular set of orthologous genes, the slope of the relationship between relative coverage and divergence from the *E. coli* gene (solid lines in figs. 3*A*–3*D* and S2, Supplementary Material online) estimates the main effect of divergence on transferability of the orthologs. Among the most stringent set of 1,295 protein-coding genes in our data set, connectivity shows a significant negative interaction with the effect of divergence on transferability (fig. 3*H* and table 2). Native expression level and functional category do not (fig. 3*I* and *J* and table 2). Examining the deviations (i.e., residuals) of each gene's effect of divergence on transferability from the value that would be expected given the gene's native expression level and COG functional category, we found a significant negative relationship between connectivity and the effect of divergence residuals (fig. 4*D*, effect estimate = −3.3 × 10^−5^, *F*_1,1294_ = 9.037, *P* = 0.0027). That is, we found a significant divergence × connectivity interaction. Like the main effect of connectivity, the power of this analysis stems largely from the translation proteins but the divergence × connectivity interaction appears to be a general characteristic of all protein-coding genes. The divergence × connectivity interaction is negative and of similar magnitude among the 61 translation proteins (fig. 4*E*; estimate = −4.0 × 10^−5^, *F*_1,192_ = 11.44, *P* = 0.0009) and the 1,102 nontranslation proteins (fig. 4*F*; estimate = −5.6 × 10^−5^, *F*_1,1101_ = 3.98, *P* = 0.0463). The results described in this and the previous paragraph do not appear to be constrained to the 1,295 genes we analyzed here. These effect estimates were quantitatively similar in analyses of the 2,653 (or 1,883) orthologous gene sets that met the less stringent criteria of including a minimum of 4 (or 10) orthologs and a standard deviation greater than 4% (or 7%) amino acid difference (supplementary table S3, Supplementary Material online), but these data sets tended to be too noisy to sufficiently power the statistical tests.

**Table 2:** Analysis of Variance Table: Biological Effects on the Divergence × Connectivity Interaction

Model Parameter	df	SSE	MSE	*F* Value	*P* Value
Connectivity	1	0.002711	0.0027113	42.56	9.87E − 11
COG functional category	20	0.000884	0.0000442	0.694	0.8357
Native expression level	1	0.000117	0.00011658	1.83	0.1764
Residuals	1,273	0.081097	6.371 × 10^−5^	—	—

Note.—Statistical model: effect of divergence ∼ connectivity + COG category + native expression level.

## Discussion

In this paper, we built on the recognition by Sorek et al. (2007) that the shotgun libraries used to generate the earliest whole prokaryotic genome sequences could be used as experimental tests of the complexity hypothesis. Sorek et al. (2007) observed across 85 finished microbial genomes that a subset of genes appeared “unclonable,” as indicated by an absence of sequencing reads spanning a gene. They then followed a bottom-up approach, experimentally investigating a modest set of particular genes from particular genomes that failed to transfer and identifying the molecular mechanism that explained each failure. Here, we focused on a top-down approach, using the shotgun libraries to estimate relative rates of transfer for large numbers of genes from large numbers of genomes. We then tested the ability of two general hypotheses—the balance and complexity hypotheses—to explain differences in the estimated transfer rates between genes and genomes. Our quantitative analysis confirmed that 1) transferability decreased with the connectivity of the transferred gene and 2) protein sequence divergence between transferred and native orthologs reduced transferability more for genes with higher connectivities than for genes with lower connectivities. The first observation is predicted by both the balance and complexity hypotheses; the second observation is predicted *only* by the complexity hypothesis (fig. 1). Thus, although our analysis is consistent with the balance hypothesis, in that the cost of protein overexpression increases with connectivity, it provides stronger support for the complexity hypothesis. Specifically, our analysis suggests that the success or failure of a transferred gene to engage in normal PPIs is an important determinant of which genes successfully undergo HGT, and that the probability of HGT failure increases via a negative (i.e., synergistic) interaction between increasing connectivity and increasing divergence.

We note that the complexity and balance hypotheses predict consequences of horizontal transfer for PPI failure and misexpression, specifically of genes native to the recipient bacterium. To test these hypotheses, we had to narrow our focus to orthologs of genes in the *E. coli* genome. Consequently, our support for the complexity hypothesis is most relevant to its role in phenomena such as the expansion of protein families and the acquisition of novel gene functions, for which the acquisition of genes with orthologous native copies is a necessary intermediate. The extent to which complexity also poses a barrier to HGT of accessory genes (such as virulence factors or antibiotic resistance genes) that are not orthologous to any native gene is also of interest but falls beyond the scope of the work we present here.

Our results highlight several of the difficulties inherent in testing the balance and complexity hypotheses experimentally: 1) connectivity and divergence are not the only biological characteristics of genes correlated with transferability, 2) many of these characteristics are also correlated with each other (supplementary fig. S5, Supplementary Material online), and 3) gene function and connectivity are poorly understood for many genes. For example, connectivity explains substantially more of the variance among all genes in their mean transferability (*R*^2^ = 0.16 *before* controlling for the correlated effects of native expression level and COG category) than among their mean transferability residuals (*R*^2^ = 0.02 *after* controlling for the correlated effects of native expression level and COG category). Connectivity also explains substantially more of the variance in mean transferability residuals among the translational proteins (*R*^2^ = 0.09), for which function and connectivity are better understood, than among the nontranslational proteins (*R*^2^ = 0.01), which include many proteins of unknown function and connectivity. Thus, our conservative decisions to control for the potentially confounding effects of native expression level and COG category and to include many poorly characterized genes in our analysis likely resulted in an *underestimate* of the contributions of connectivity and divergence to transferability.

Even in a less conservative analysis that includes only the translational proteins and does not control for the correlated effects of native expression level, connectivity explains only 31% of the variance in transferability (i.e., *R*^2^ = 0.31) and 16% of the variance in the effect of divergence on transferability, leaving most of the variance unexplained. Other than the fact that genomic data are inherently noisy, in part because genomes still contain many poorly characterized genes, we do not know what explains the remaining variance. We examined more closely the set of proteins with more than 80 PPIs to try to identify differences between the proteins that fall substantially above and substantially below the regression lines in figure 4*A* and *D*. Among this set of highly connected genes, the genes that were more versus less transferable than expected did not differ in their identities, functions, lengths, number of disordered regions, or number of identified orthologs in our collection. The ten most transferable genes among this set (ribosomal subunit proteins S4, S8, S11, S15, L17, L28, L32, and L33 and RNA polymerase subunits alpha and omega) are, to our eyes, indistinguishable from the ten least transferable genes among this set (ribosomal subunit proteins S7, S10, S12, S20, L3, L4, L11, and L34 and RNA polymerase subunits beta and beta′). However, the differences in mean transferability and in the effect of divergence on transferability (more specifically, the residuals in fig. 4*A* and *B*) did depend on the distribution among the orthologs in each gene set of their divergence from the *E. coli* gene. Genes whose orthologs were, on average, more divergent from the *E. coli* gene copy were less transferable (effect of mean divergence on the residuals in fig. 4*A*: estimate = −0.0045, *F*_1,52_ = 6.781, *P* = 0.012, adjusted *R*^2^ = 0.1). In contrast, genes whose orthologs exhibited higher variation in divergence from the *E. coli* gene copy were more transferable (effect of the standard deviation of divergence on the residuals in fig. 4*A*: estimate = 0.0064, *F*_1,52_ = 9.046, *P* = 0.004, adjusted *R*^2^ = 0.13). Sets of gene orthologs with higher variation in their divergence from *E. coli* also exhibited weaker (more positive) effects of divergence on transferability (effect of the standard deviation of divergence on the residuals in fig. 4*B*: estimate = 0.0004, *F*_1,52_ = 11.11, *P* = 0.0016, adjusted *R*^2^ = 0.16). These patterns indicate that there are effects of divergence on transferability beyond the divergence × connectivity interaction predicted by the complexity hypothesis. Because the data for this analysis were originally gathered for a different purpose, this “experiment” was unavoidably unbalanced. For instance, we could not ensure a similar distribution of divergence values among genes with low and high PPIs. As a result, the main effects of divergence contributed to the difficulties inherent in our test of the complexity hypothesis. We note, however, that these effects of the mean and standard deviation of divergence were apparent only when we limited our focus to genes with more than 80 PPIs. None of these effects was apparent in the full data set.

The difficulties inherent in testing the balance and complexity hypotheses were also highlighted by a previous experimental test (Acar Kirit et al. 2020) that did not detect a statistically significant main effect of connectivity on transferability (as defined in fig. 1*C*), despite the use of a more precise measure of transferability than ours. Acar Kirit et al. (2020) examined a substantially smaller number of genes (44 compared with 1,295) from a smaller number of donor genomes (1 compared with ≥16 for each gene). Thus, the difference in outcome is likely to have resulted from the limited statistical power of their smaller data set to detect a main effect of connectivity on transferability. Although their precise fitness assays enabled the detection of fitness costs imposed by two biological characteristics of genes that we did not detect, increasing the number of disordered regions and the length of transferred proteins, their use of only a single donor genome prevented an examination of the statistical interaction between connectivity and divergence in their effects on transferability (as in fig. 1*D*). Because the detection of interaction effects requires more statistical power than the detection of main effects, our ability both to differentiate between the two hypotheses and to support the complexity hypothesis was likely only possible because our data set was compiled from 74 whole genome shotgun libraries.

Indeed, our strongest support of the complexity hypothesis comes from the *consistency* of the interaction between divergence and connectivity among both proteins with functions in translation and proteins with other functions (fig. 4). The complexity hypothesis was proposed to explain the observation from comparative data that HGT has happened less often among informational genes, like the subunits of ribosomes and polymerase complexes, than among operational genes, like enzymes (Rivera et al. 1998; Jain et al. 1999). The complexity hypothesis posits that the observed difference in the rate of HGT resulted not from the difference in function between informational and operational genes but rather from the large difference in the connectivities of these different types of genes. Its central prediction is that increases in connectivity and the divergence between donor and recipient orthologs will interact synergistically to reduce transferability. In short, highly divergent genes with many PPIs will exhibit the lowest rates of HGT, regardless of function. Thus, our finding that connectivity interacts with divergence to reduce transferability, not only among the translation (i.e., informational) genes but also among nontranslation (i.e., noninformational) genes, supports both the central prediction of the complexity hypothesis and its underlying logic that informational genes exhibit lower rates of HGT specifically because they are highly connected, not because they perform an informational function.

Our results confirm the central predictions of both of the two nonexclusive hypotheses used to explain why transferability should decrease with connectivity. We provide the strongest support for the role of PPI failure among divergent orthologs of highly connected genes (the complexity hypothesis). Together, our results support the emerging “rule” that deleterious interactions among protein partners in bacteria may govern the frequency with which individual genes successfully undergo HGT. Given that HGT is a major contributor to bacterial genome evolution, our work suggests that the complexity hypothesis may shape phenotypic diversity, drive the expansion of protein families, and affect the evolution of new phenotypes, new metabolic pathways, and new species in bacteria.

## Materials and Methods

### Data Sources

The complete set of whole genome shotguns and assembled genomes used in this work is described in supplementary table S1, Supplementary Material online.

We obtained whole genome shotgun reads from NCBI's trace archive (ftp://ftp.ncbi.nih.gov/pub/TraceDB). We included all of the shotguns examined in Sorek et al. (2007) except *Candidatus* Koribacter versatilis Ellin345, whose shotgun reads were no longer available in the trace archive.

We obtained complete bacterial genome sequences from NCBI's microbial genome database (https://www.ncbi.nlm.nih.gov/genome/microbes/). From these sequence files, we determined the protein sequence, start position, end position, and length for each coding sequence in each genome.

We obtained protein interaction data from the STRING database version 11.5 (http://string-db.org). We downloaded two files containing detailed confidence scores associated with evidence of PPIs in *E. coli* strain K12 substrain MG1655, one including evidence of physical interactions only (511145.protein.physical.links.detailed.v11.5.txt) and the other including evidence of both physical and nonphysical interactions (511145.protein.links.detailed.v11.5.txt). For each gene, we extracted the confidence score from the experimental column of these files. Confidence scores range from 0 (low confidence) to 999 (high confidence). We considered pairs of proteins to be interacting if their confidence score, based on experimental evidence only, was greater than a particular threshold value. Our statistical tests of the balance and complexity hypotheses produced qualitatively and quantitatively similar conclusions regardless of the choice to count only physical interactions or both physical and nonphysical (as represented in the STRING database) or the choice of confidence score cutoff between 200 and 800 (analyses not shown). For the analysis described here, we counted both physical and nonphysical interactions and used an intermediate confidence score threshold of 500.

We obtained expression data for the native copy of each gene in *E. coli* from the ASAP database (https://asap.genetics.wisc.edu/asap/experiment_data.php). We downloaded data from two replicate (PALSP49 and PALSP50) calibrated microarray experiments conducted by Allen et al. (2003) on *E. coli* strain K12 MG1655, grown to log phase in LB liquid medium at 37 °C. For each gene, we calculated native expression level as the mean of the estimated transcript copy number across the two replicate experiments.

We obtained the functional category of each gene from the COG database at https://www.ncbi.nlm.nih.gov/research/cog/ (Tatusov et al. 1997; Galperin et al. 2015).

We obtained the number of disordered regions for all of the genes with at least 80 PPIs using the web service GlobPlot at http://globplot.embl.de (Linding et al. 2003).

### Gene Coverage in the Shotguns

The coverage, *c_ij_*, of gene *i* in the whole genome shotgun of species *j* was calculated as the number of plasmid inserts in shotgun *j* that contain gene *i* in its entirety. Coverage values were determined by mapping the paired reads from each whole genome shotgun to the corresponding assembled genome using the BWA-SW algorithm from the Burrows–Wheeler Aligner (Li and Durbin 2010). A small minority of reads mapped to more than one location. We identified and eliminated most of the incorrectly mapped reads by requiring a phred-scaled map quality score greater than 150 and a distance between paired reads of fewer than 100,000 bases. For the few multiply-mapped reads that remained, one of the mapping locations was chosen at random. Read pairs for which one read mapped upstream of gene *i* and one read mapped downstream of gene *i* were counted toward the coverage of gene *i*. Read pairs that entirely spanned more than one gene were counted toward all of the spanned genes. Although we mapped read pairs to all of the replicons that comprised each genome, only genes contained on large replicons (i.e., chromosomes) were included in downstream analyses. We ignored genes on plasmids because their biology differs from genes on chromosomes (e.g., their copy number is often higher) and because their dramatically lower gene content resulted in dramatically higher variance in coverage and, consequently, much less ability to correct for the known biases in the data that we describe below.

### Effect of Gene Length on Coverage

To control for the bias against long genes, we calculated for each genome *j* a likelihood of observing a gene of a particular length, *l*, given the actual distribution of cloned fragment lengths, *f_j_*, that comprised whole genome shotgun *j*.

For an individual cloned fragment of length *x*, the probability that it contained the entirety of a gene of length *l* is proportional to the difference in the lengths between the cloned fragment and the gene, if the cloned fragment is at least as long as the gene, or 0 otherwise:


(1) $$\begin{array}{c} \;p(l|x)∼x+1-l\;\;\;\;\;\;\;\;\;if\;\;x\geq l\\ \;p(l|x)=0\;\;\;\;\;\;\;\;\;\;\;\;\;\;\;\;\;\;\;\;if\;\;x<l. \end{array}$$

For each genome *j*, the likelihood of observing each gene, *g_ij_*, of length, *l_ij_*, given the distribution of cloned fragment lengths, *f_j_*, is then calculated by summing these individual probabilities over all of the observed cloned fragment lengths *x_j_* in the shotgun of genome *j*:


(2) $$L(g_{ij}|f_{j})=\underset{x_{j}}{\sum }\;p(l_{ij}|x_{j}).$$

For each genome *j*, the bias against long genes, *b_j_*(*l*), was then calculated as the likelihood of observing a gene of length *l* relative to that of the most likely (i.e., the shortest) gene in the same genome:


(3) $$b_{j}(l)=L(l|f_{j})/L(\min(l_{j})|f_{j}).$$

For each genome, coverage values for each gene were adjusted by the length biases calculated for that genome, so that the data used for all downstream analysis were of the form:


(4) $$c_{ij}^{*}=\frac{c_{ij}}{b_{j}(l_{ij})}$$

### Effect of Gene Position on Coverage

To control for the long-range positional biases, we fit the adjusted coverage data from individual genomes to a sine curve using the *lm* function in R (figs. 2*C*, 2*F*, and S1*D*, Supplementary Material online). For gene *i* in genome *j*, we calculated relative coverage, *c_ij_*, by dividing its length-adjusted coverage value, ${\hat{c}}_{ij}$, by the fitted value at that position in the genome, *E*(c_ij_*|position*_ij_*):


(5) $${\hat{c}}_{ij}=c_{ij}^{*}/E(c_{ij}^{*}|{\mathrm{position}}_{ij})$$

### Effect of Divergence on Coverage

For each protein-coding gene in the *E. coli* K12 genome, we identified the coding sequences in each shotgun library that were reciprocal best hits to the *E. coli* K12 gene using BLASTp (Altschul et al. 1990). Coding sequences that were not reciprocal best hits could not be identified as the most likely ortholog of a particular coding sequence in *E. coli* K12 and were, therefore, excluded from downstream analyses. The protein sequences of reciprocal best hits were aligned to the *E. coli* K12 gene using the software ProbCons (Do et al. 2005), and the alignments were used to calculate protein divergence as % amino acid difference from the *E. coli* K12 ortholog. For each of the resulting sets of orthologous genes, we used the *lm* function from the *stats* package in R (version 4.1.2) to determine the best fit linear relationship between the relative coverage of each ortholog in the set and its divergence from the *E. coli* ortholog.

### Statistical Tests of the Balance and Complexity Hypotheses

We used the *lm* function from the *stats* package in R (version 4.1.2) to investigate the effects of connectivity and divergence on the transferability into *E. coli* of orthologs of the protein-coding genes in the *E. coli* genome (as illustrated in fig. 1). For each *E. coli* gene, we estimated mean transferability (the *y* axis in fig. 1*C*) as the mean relative coverage across all orthologs of the gene. We measured the effect of divergence on transferability (i.e., the main effect of divergence; *y* axis in fig. 1*D*) as the slope of the linear relationship between relative coverage and divergence for all orthologs of the gene (as described above in Effect of Divergence on Coverage). In essence, this statistical approach considers connectivity and divergence as fixed effects and the identity of the *E. coli* ortholog as a random effect. The experimental units are the sets of gene orthologs and the analysis considers the individual orthologs in each set to be repeated measures of a particular orthologous gene along a divergence gradient.

### Power Analysis

A visual examination of the data in supplementary figure S2, Supplementary Material online, revealed that the highest positive and lowest negative estimates of the effects of divergence on relative coverage were most common among genes for which the set of identified orthologs spanned only a narrow range of divergence values or for which we identified only very few orthologs. In addition, the smaller sets of orthologous genes often included at least one gene with an exceptionally high divergence (>80% amino acid difference) from the *E. coli* ortholog, suggesting that these sets included donor genes that were not true orthologs. To examine the sensitivity of our statistical tests to the noisy data that resulted from these issues, we repeated the analysis described above, requiring that each set of orthologous genes includes at least *X* orthologs and exhibits a standard deviation greater than *Y*% amino acid difference, where *X* and *Y* were both varied between 2 and 22. For each pair of *X* and *Y*, we estimated the number of genes that met both requirements, the main effect of connectivity on mean transferability, and the *F* statistic and *P* value associated with the statistical test of the effect (supplementary fig. S3, Supplementary Material online). For each pair of *X* and *Y*, we also estimated the connectivity × divergence interaction effect (i.e., the effect of connectivity on the main effect of divergence; see fig. 1*D*), as well as the *F* statistic and *P* value associated with the statistical test (supplementary fig. S4, Supplementary Material online).

## Supplementary Material

evad089_Supplementary_Data

## Data Availability

The data are publicly available and described in supplementary table S1, Supplementary Material online. The analysis pipeline consists of programs written in Python 3.6 and R version 4.1.2 (R Development Core Team 2013), and UNIX shell scripts that call those programs, BEDTools (Quinlan and Hall 2010), and SAMtools (Li et al. 2009). All of our code and scripts are publically available at https://bitbucket.org/cburch/burch-et-al-2023.
